# Morphometric analysis of immunoselection against hyperploid cancer cells

**DOI:** 10.18632/oncotarget.5400

**Published:** 2015-10-15

**Authors:** Norma Bloy, Allan Sauvat, Kariman Chaba, Aitziber Buqué, Juliette Humeau, José Manuel Bravo-San Pedro, Jack Bui, Oliver Kepp, Guido Kroemer, Laura Senovilla

**Affiliations:** ^1^ Equipe 11 Labellisée Ligue contre le Cancer, Centre de Recherche des Cordeliers, INSERM U 1138, 75006 Paris, France; ^2^ Université Paris Sud, Faculté de Médecine, 94270 Kremlin Bicêtre, France; ^3^ Université Paris Descartes, Sorbonne Paris Cité, 75006 Paris, France; ^4^ Université Pierre et Marie Curie, 75006 Paris, France; ^5^ Metabolomics and Cell Biology Platforms, Gustave Roussy Comprehensive Cancer Institute, 94805 Villejuif, France; ^6^ Department of Pathology, University of California San Diego, CA 92093, USA; ^7^ Pôle de Biologie, Hôpital Européen Georges Pompidou, AP-HP, 75015 Paris, France; ^8^ Karolinska Institute, Department of Women's and Children's Health, Karolinska University Hospital, 17176 Stockholm, Sweden

**Keywords:** morphometric analysis, ploidy, ER stressi, mmunoselection

## Abstract

An at least transient increase of ploidy, usually by whole genome duplication, is a frequent event in oncogenesis, explaining the cytogenetic features of at least 40% of solid cancers. Here, we show that fibrosarcomas induced by the carcinogen methylcholanthrene (MCA) are distinct with respect to their ploidy status when they arise in immunocompetent wild type *versus* severely immunodeficient *Rag2*^−/−^*γc*^−/−^ mice. MCA-induced fibrosarcomas are particularly hyperploid if they develop in an immunodeficient setting, correlating with higher DNA content, increased nuclear surface, as well as hyperphosphorylation of eukaryotic initiation factor 2` (eIF2`), a biomarker indicating endoplasmic reticulum (ER) stress. Upon transfer of such cells into wild type mice, such hyperploid, ER-stressed cells (that originated in *Rag2*^−/−^*γc*^−/−^ mice) fail to proliferate and actually induce a protective anticancer immune response. In contrast, such cells do form tumors in *Rag2*^−/−^*γc*^−/−^ recipients (which lack T, B and NK cells) as well as in *Rag2*^−/−^ recipients (which only lack T and B lymphocytes) and conserve their hyperploidy as well as eIF2` hyperphosphorylation. To measure these parameters, we developed a morphometric analysis tool that is applicable to immunohistochemistry of formaldehyde-fixed, paraffin-embedded tissues. This software automatically identifies and quantifies the surface of nuclei and determines the intensity of eIF2` phosphorylation within a perinuclear region of interest. Comparative analyses performed on cultured cells and tissue sections validated the accuracy of this method, which can be used to investigate ploidy and ER stress in cancers *in situ*.

## INTRODUCTION

Approximately one fifth of human cancers are characterized by higher order of aneuploidy (i.e., a near-to-polyploid chromosomal content) [1]. Aneuploidy, which is a close-to-constant feature of cancer, may arise as a consequence of a whole-genome doubling event followed by the missegregation and progressive loss of chromosomes [2]. Multiple lines of evidence support this pathway. First, extensive whole-genome characterization of multiple solid cancers revealed that approximately 40% among them, including those with a near-to-diploid DNA content, have experienced at least one event of whole-genome doubling during their evolution [3–5]. Second, tetraploid cells, which are cells that have undergone one event of genome duplication, are found in early-stage breast, cervical and colorectal carcinomas, as well as in pre-malignant lesions like Barrett esophagus [1]. At the experimental level, it has been demonstrated that tetraploid, but not diploid, mammary or colorectal epithelial cells lacking p53 can form tumors upon inoculation into immunodeficient mice [6–8]. Chemopreventive agents such as resveratrol and aspirin, which reduce the frequency of colorectal cancer, are known to reduce the frequency of tetraploid cells in the intestine from genetically cancer-prone mice [9]. Hence, hyperploidy, an increase in the number of chromosomes is a common feature of oncogenesis that is causally involved in the molecular etiology of oncogenesis [10].

Although cancer has been traditionally conceived as a cell-autonomous genetic and epigenetic disease [11], it has been recently recognized that this disease has also an immunological dimension [12–14]. Thus, cancers can only develop in the context of failing immunosurveillance. As a result, tumors generally are more frequent and progress more quickly in immunodeficient than in immunocompetent mice [15]. Moreover, tumors that have arisen in immunodeficient mice generally fail to proliferate upon their transplantation into immunocompetent mice [15–17]. In the unlikely event that cancer cells stemming from immunodeficient mice finally produce tumors in normal recipients, the malignant cells contained therein differ from the initial transplant, a process that is called ‘immunoediting’ [18]. Immunoedited cancer cells either lose the expression of tumor-associated antigens, TAA, (and hence reduce their ‘antigenicity’) [19] or that of danger-associated molecular patterns (DAMPs) that can stimulate immune reactions (and hence decrease their ‘adjuvanticity’) [20, 21]. In addition, immunoedited cancer cells may have acquired the capacity to actively suppress the anticancer immune response [22]. Irrespective of the precise molecular mechanisms, pre-malignant cancer cells must orchestrate their escape from immunosurveillance to generate full-blown neoplasias.

In addition to cell-autonomous control mechanisms that avoid or abort the process leading to hyperploidization (such as those involving tumor suppressor genes) there is clear evidence that tetraploid cells are normally eliminated by the immune system. Thus, cells that have been rendered tetraploid *in vitro* and then are injected into mice, only induce tumors when the cellular immune system is dysfunctional due to genetic defects causing the ablation of T cells (such as the *nu/nu* mutation or the knockout of the Rag2 recombinase) or following the injection of antibodies that deplete CD8^+^ cytotoxic T lymphocytes [23]. The capacity of the immune system to recognize and destroy tetraploid cells has been explained by the fact that such cells develop an endoplasmic reticulum (ER) stress response, thereby stimulating the exposure of calreticulin on the cell surface [7, 8, 23]. When present on the plasma membrane, calreticulin serves as an ‘eat-me’ signal [24], hence facilitating the recognition of cancer by myeloid cells [25, 26], the engulfment of portions of tumor cells by immature dendritic cells [26], and cross-presentation of TAAs to cytotoxic T lymphocytes. The underlying mechanism of calreticulin exposure involves the phosphorylation of eukaryotic initiation factor 2α (eIF2α) [27, 28], which is a major sign of ER stress. Accordingly, hyperploid cells exhibit the hyperphosphorylation of eIF2α, coupled to the increased surface exposure of calreticulin [23]. As a consequence, phosphorylation of eIF2α, which can be detected with phospho-neoepitope-specific antibodies, constitutes a biomarker of cancer cell adjuvanticity [29]. Importantly, when tetraploid cells are injected into immunocompetent mice, cancers occasionally develop with delayed kinetics. Reanalysis of the observed tumors indicates that they reduce ploidy, as well as eIF2α phosphorylation and calreticulin exposure. These results underscore the importance of eIF2α phosphorylation for the induction of anticancer immune responses against hyperploid cells.

The present study has been designed with a dual scope, namely (i) to develop an automated image analysis system that allows to measure ploidy and eIF2α hyperphosphorylation on tissue sections and (ii) to apply this technology to the question whether carcinogen-induced cancers arising in T cell-deficient mice exhibit differences in ploidy and eIF2α phosphorylation with respect to cancers developing in immunocompetent animals.

## RESULTS AND DISCUSSION

### Comparison of diploid and hyperploid tumor cells by immunohistochemical methods

**Figure 1:**
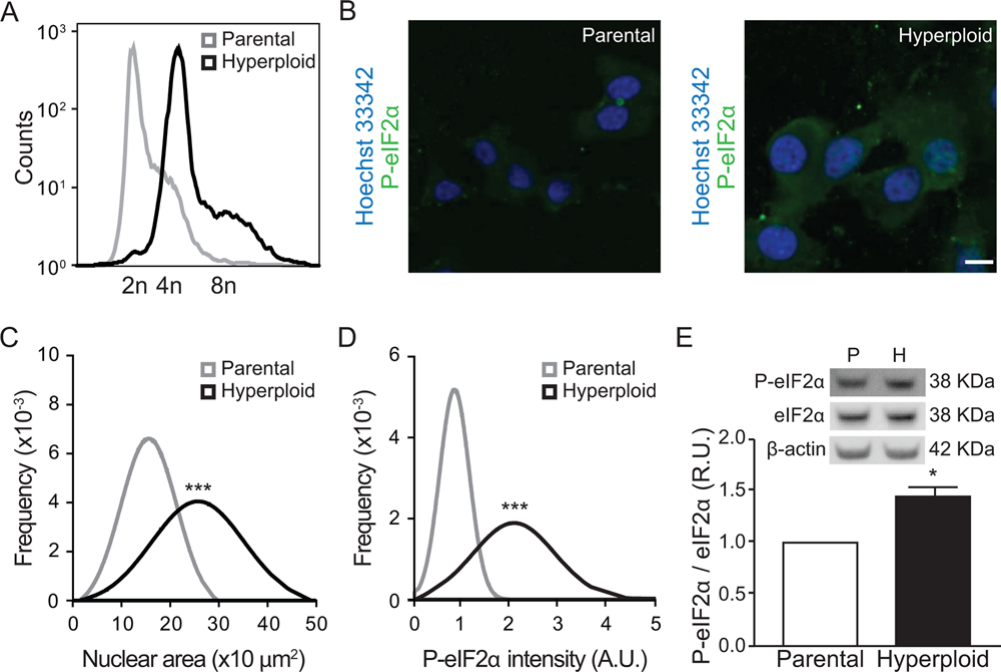
Linkage between hyperploidy and ER stress in CT26 cell line **A.** CT26 parental cells and CT26 hyperploid clones were fixed and stained with propidium iodide and their chromosomal content was detected by FACS. The plot is representative of seven independent assessments, which yielded similar results. **B–D.** Parental and hyperploid CT26 cells were processed for the software-assisted, (immuno)fluorescence-based detection of Hoechst 33342 and phosphorylated eIF2α. Representative images are reported in (B) (scale bar, 20 μm; *n* = 3) and quantitative data for normal distribution of nuclear area (C) and P-eIF2α intensity (D) were obtained using the MetaXpress software. Alternatively, phosphorylated and total eIF2α were assessed by quantitative immunoblotting (*n* = 3) **E.** Statistical analysis was performed with one-tailed Student's *t* tests. Error bars indicate SEM. *p < 0.05, ***p < 0.001 as compared with the parental cell line.

CT26 colon cancer cells are normally close-to-diploid, yet can be rendered hyperploid by transient exposure to the reversible microtubular inhibitor nocodazole, followed by cytofluorometric purification of cells incorporating high levels of the chromatin stain Hoechst 33342 [30]. By this method, stable hyperploid clones can be obtained. As compared to parental CT26 cells, such hyperploid derivatives exhibit elevated chromosome content, as detectable by fluorescence-activated cell sorter, FACS, analysis after staining DNA from trypsinized and permeabilized cells with propidium iodide (Fig. 1A). A similar result was obtained upon microscopic observation of adherent cells *in situ*, after staining with Hoechst 33342, revealing an increase in the nuclear area in hyperploid cells (Fig. 1B, 1C). Simultaneous immunofluorescence detection of phosphorylated eIF2α (on serine 51, P-eIF2α) unveiled an ER stress response that was exacerbated in hyperploid cells (Fig. 1B, 1D) and could be confirmed by immunoblot detection of P-eIF2α protein (Fig. 1E).

**Figure 2:**
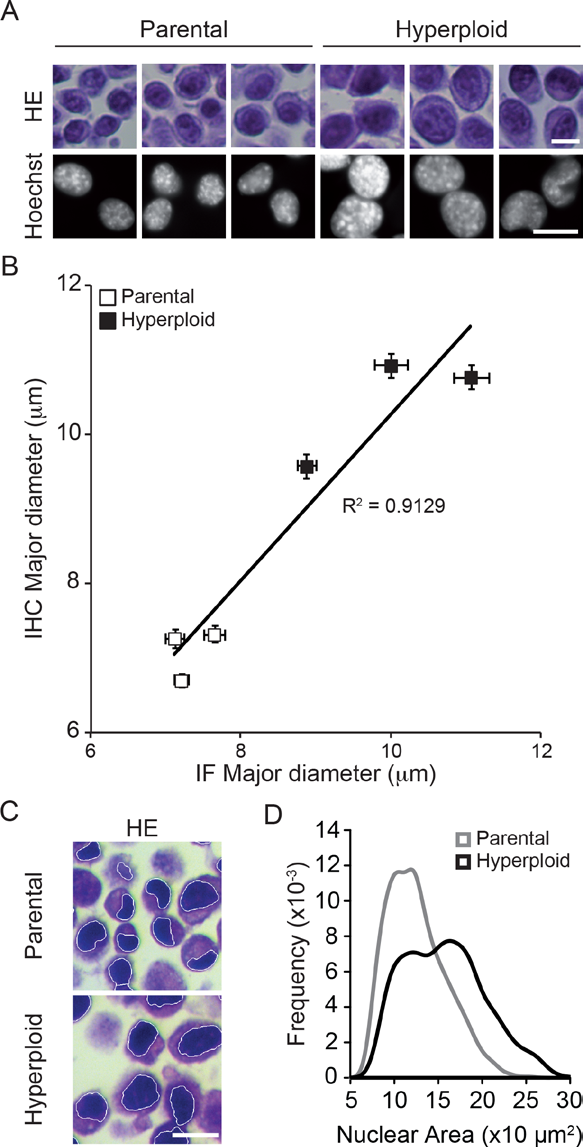
Nuclear diameter as an indirect measurement of ploidy in HES sections **A, B.** Murine colon carcinoma CT26 parental and hyperploid clones were subjected both to fluorescence microscopy upon Hoechst 33342 staining and to hematoxilin/eosin (HE) staining upon inclusion into paraffin pellets. Representative pictures are shown in (A) and the correlative quantification in (B). **C, D.** Morphometric analysis were performed with the algorithm developed in R on the nuclear area after segmentation of the hematoxylin stained nuclei (C), and the nuclear area of the parental or hyperploid clones were automatically quantified (D). Scale bar, 20 μm. Results are representative of 6 different clones.

**Figure 3:**
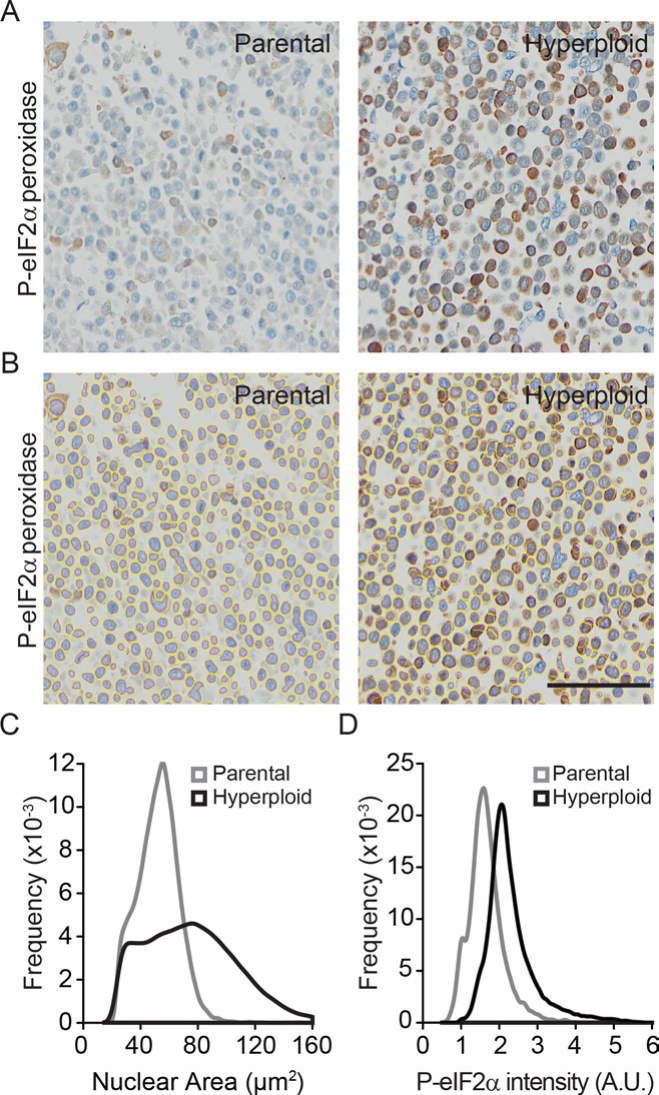
Algorithm validation for the nuclear size and P-eIF2α intensity quantifications in P-eIF2α tissue staining **A–D.** Morphometric analysis were carried out on CT26 parental and hyperploid clones to quantify the nuclear area (Hematoxylin II plus Bluing reagent staining) and P-eIF2α (peroxidase staining) intensity of immunohistochemistry stained pellet sections (A) after segmentation (B) Scale bar, 50 μm. Quantifications for nuclear area (C) and P-eIF2α intensity (D) were obtained using the algorithm developed in R. Results are representative of 6 different clones.

In the next step, we wondered whether the increase in nuclear size and eIF2α phosphorylation could also be detected by immunohistochemical methods. Pellets of parental and hyperploid CT26 cells that had been trypsinized and spun down by centrifugation were treated similarly as biopsies and hence paraffin embedded, stored at −20°C and subjected to deparaffinization before hematoxylin eosin (HE) staining (Fig. 2) or immunohistochemical detection of P-eIF2α (Fig. 3). Comparative HE staining of several clones revealed a similar hyperploidy-associated increase in the diameter of nuclei (which stain intensely with hematoxylin) as we had detected by Hoechst 33342 staining of cultured cells *in situ* (Fig. 1B, 2A, 2B). This result was initially obtained by manually measuring the largest diameter of individual nuclei. Morphometric analysis of the HE-stained samples corroborated a hyperploidy-associated augmentation of the nuclear area (Fig. 2C, 2D). Immunohistochemical detection of P-eIF2α also confirmed the hyperphosphorylation of this ER stress-associated protein in hyperploid cells. This result was obtained by means of an automated procedure in which sections stained by immunohistochemistry were scanned in a specialized microscopic device (Fig. 3A) and subjected to segmentation to distinguish cells and nuclei (Fig. 3B, 3C). Finally, a perinuclear area was defined for quantitating the intensity of the P-eIF2α-dependent signal (Fig. 3D). Altogether, these data indicate that the characteristics of hyperploidy (increased nuclear diameter or surface and hyperphosphorylation of eIF2α) can be measured in paraffin-embedded tissues that are subjected to HE staining or P-eIF2α-specific immunohistochemistry.

### Hyperploidy and eIF2α hyperphosphorylation of non-immunoselected tumors

**Figure 4:**
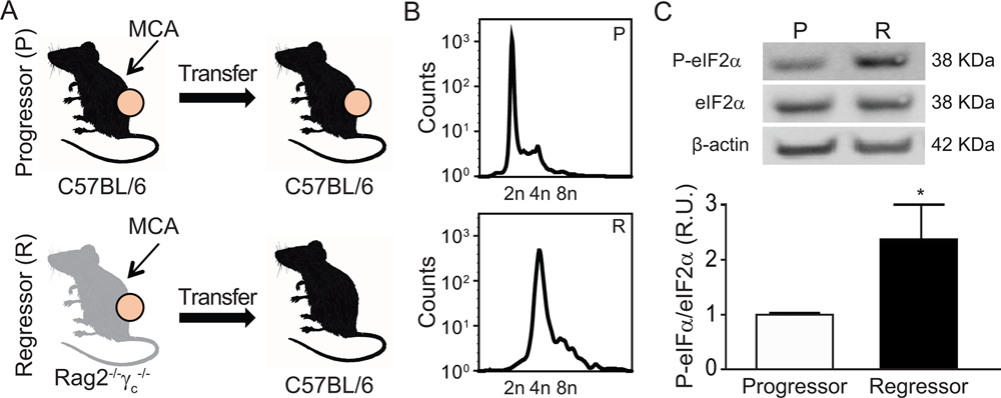
Characterization of the MCA-induced fibrosarcomas developed into immunocompetent *vs* immunodeficient mice Methylcholanthrene (MCA)-induced fibrosarcomas were developed into immunocompetent C57BL/6 or immunodeficient *Rag2^−/−^γ_c_^−/−^* mice. Generated MCA-fibrosarcoma cell lines were transplanted into syngenic C57BL/6 mice. A cell line was considered “Progressor” or “Regressor” if it was able to growth in > or < 50% of C57BL/6 injected mice, respectively **A.** Progressor and regressor cell lines were characterized by their DNA content by FACS (representative plots of five independent experiments) **B.** and phosphorylation levels of eIF2α by immunoblotting (*n* = 3) **C.**

Methylcholanthrene (MCA)-induced fibrosarcomas from immunocompetent C57BL/6 mice can be transplanted into secondary C57BL/6 hosts and hence exhibit a ‘progressor’ phenotype. In contrast, fibrosarcomas that have been induced by MCA in immunodeficient *Rag2^−/−^*γc*^−/−^* mice (and which hence have not undergone any immunoselection due to the absence of B, T and NK lymphocytes) usually fail to grow - or regress after initial growth - upon transplantation in immunocompetent C57BL/6 recipients. Such MCA fibrosarcoma cells are considered to have a ‘regressor’ phenotype (Fig. 4A). As compared to progressors, regressor cells contained a higher DNA content (Fig. 4B) and exhibited an elevated eIF2α phosphorylation (Fig. 4C), supporting that the absence of immunoselection favors the outgrowth of hyperploid, ER-stressed tumor cells.

**Figure 5:**
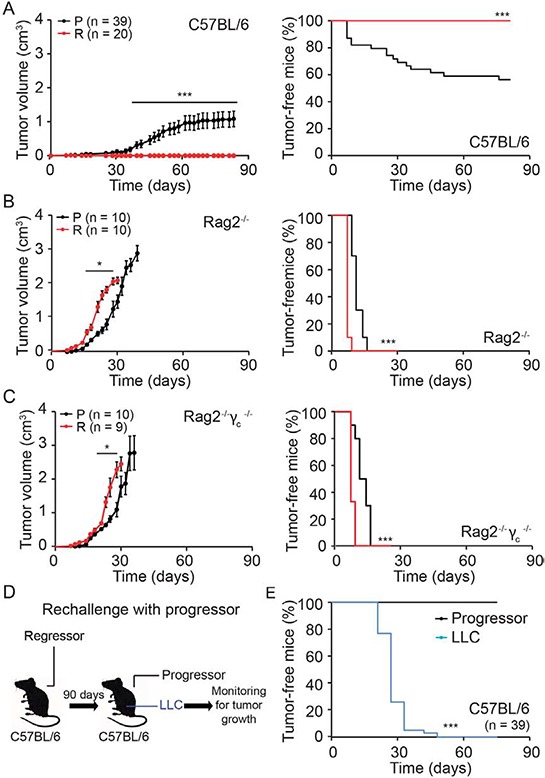
Immunogenicity of regressor cell line **A–C.** Progressor and regressor cells were inoculated into C57BL/6, *Rag2^−/−^ or Rag2^−/−^γ_c_^−/−^* mice. **D, E.** C57BL/6 mice that stayed tumor free for three month after regressor cell line injection were reinjected with either progressor cells or with unrelated murine LLC cells. Tumor growth (left panels in A-C) and incidence (right panels in A-C and E) were routinely monitored. Tumor growth curves (on the left) were analyzed with one-tailed Student's *t* test, whereas tumor incidence (right graphs, illustrated with Kaplan-Meier curves) was compared by log rank test. Error bars indicate SEM. *p < 0.05, ***p < 0.001 as compared with the progressor cell line.

In a series of control experiments, we confirmed that progressor but not regressor cell lines grew after subcutaneous inoculation into immunocompetent C57BL/6 mice (Fig. 5A). In sharp contrast, there was no major difference in tumor growth and the incidence of cancer between progressor and regressor cells injected into immunodeficient *Rag2*^−/−^ mice, which lack B and T lymphocytes due to the absence of the Rag2 recombinase (Fig. 5B), or *Rag2*^−/−^*γc*^−/−^ mice, which, in addition to B and T lymphocytes, also lack NK cells (Fig. 5C). Immunocompetent C57BL/6 mice that had been inoculated with regressor cells became immune to subsequent challenge with progressor cells, although they remained susceptible to the growth of the unrelated Lewis lung cancer (LLC) (Fig. 5D, 5E).

Altogether, these results support the idea that hyperploid, ER-stressed cancers arise preferentially in the context of an immunodeficiency. In other terms, hyperploid cells are usually eliminated by the adaptive (Rag2 gamma c-dependent) immune system.

### Immunohistochemical comparison of non-selected and immunoselected cancers

**Figure 6:**
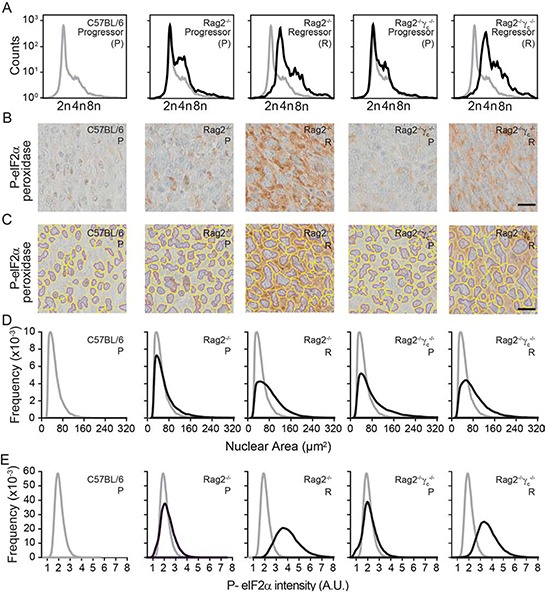
Morphometric analysis of ploidy and eIF2α phosphorylation on tumor tissue sections Progressor and regressor tumors recovered from C57BL/6 mice, *Rag2^−/−^ mice or Rag2^−/−^γ_c_^−/−^* mice were cultured *in vitro* and their ploidy was determined by cytofluorometry **A.** Alternatively, histological sections submitted to immunohistochemical staining for the detection of eIF2α phosphorylation **B–E.** Scale bar, 20 μm. Representative pictures are shown in (B) Morphometric analysis was carried out with the algorithm developed in R on the segmented images tumor sections (C), to quantify the nuclear area (D) and the phosphorylation of eIF2α (E) Results are representative of 97 recovered tumors.

To further support the conclusion that immunodeficiency is compatible with the survival of hyperploid, ER-stressed cancer cells, we dissociated tumors that arose after inoculation of progressor or regressor cells into wild type, *Rag2*^−/−^ or *Rag2*^−/−^*γc*^−/−^ mice, cultured the cells for one week (to eliminate stromal cells) and then performed cytofluorometric analyses to determine their DNA content. This procedure revealed that regressor cell lines conserved their hyperploid phenotype after *in vivo* passage through immunodeficient mice (Fig. 6A).

Freshly excised tumors were also paraffin-embedded and later processed for immunohistochemical detection of nuclei (which were counterstained with Mayer's hematoxylin in blue) and eIF2α phosphorylation (which was revealed by peroxidase and 3,3′-diaminobenzidine as a brown pigment). Using the morphometric analysis that we developed (see Materials and Methods) the corresponding microscopic scans (Fig. 6B) could be correctly segmented to detect nuclear contours (Fig. 6C), followed by calculation of the nuclear area (Fig. 6D) and quantitation of the intensity of the perinuclear eIF2α phosphorylation (Fig. 6E).

Altogether, these results indicate that the method that we developed for the morphometric analysis of ploidy and ER stress can be applied to tumor tissue sections. This method reveals that hyperploid, ER-stressed cells can only persist in an immunodeficient context.

### Concluding remarks

The present work describes an automatic software that is provided without any charge to the research community (https://fileshare.gustaveroussy.fr:443/easyshare/fwd/link=lMfqvir41hZeTLfQFT2QGD) and that allows for the quantitation of nuclear surface (which correlates with ploidy) as well as that of eIF2α phosphorylation within the cytoplasm of tumor cells subjected to fixation, paraffin embedding and immunohistochemistry. Using this method, we could show that tumors arising in severely immunodeficient mice following exposure to the carcinogen MCA are hyperploid and exhibit eIF2α hyperphosphorylation as compared to tumors induced in immunocompetent mice. Upon their injection into immunocompetent recipients such hyperploid, ER-stressed cancer cells do not form tumors and actually induce an efficient and specific anticancer immune response.

Although the aforementioned morphometric analyses turn out to be useful, they should be used while taking into account several caveats. First, nuclear size correlates with DNA content, yet is not equivalent to DNA content, because chromatin can exist in distinct degrees of compaction and condensation. Second, eIF2α hyperphosphorylation is not the sole sign of ER stress, and other potential markers (such as the presence of the spliced XBP1 variant in the nucleus of the cells or the presence of ATG6 in the Golgi apparatus or in the nucleus) must be explored in future studies. Third, morphometric analyses cannot easily distinguish stromal and malignant cells, meaning that the area of interest on which the analysis is performed must be clearly defined, ideally by a trained pathologist.

Irrespective of these limitations, the morphometric analysis tools developed here will be useful for quantitative cell-by-cell measurements that will allow for the identification of rare populations, as well as the correlation of distinct biomarkers across distinct areas of the same tumor, contributing to the description of tumor cell heterogeneity. Moreover, the morphometric tools are amenable to automation for the characterization of large numbers of tumors including in the context of tissue microarrays. In summary, we believe that the techniques described in this type, in particular the appropriate image analyses tools, will facilitate future immuno-oncological studies by measuring nuclear size as a surrogate marker of ploidy and perinuclear eIF2α phosphorylation as a surrogate maker of ER stress on a per-cell basis.

## MATERIALS AND METHODS

Unless otherwise indicated, media and supplements for cell culture were purchased from Gibco-Invitrogen (Carlsbad, CA, USA), plasticware from Corning B.V. Life Sciences (Schiphol-Rijk, The Netherlands), and chemicals from Sigma-Aldrich (St Louis, MO, USA).

### Antibodies

Rabbit polyclonal antibody against phospho-eIF2α (32157) and mouse monoclonal antibody against β-actin were purchased from Abcam (Cambridge, UK). Rabbit polyclonal antibodies against, eIF2α (9722) and anti-phospho-eIF2α (3597) were from Cell Signaling Technology (Danvers, USA).

### Cell lines and culture conditions

All cell lines were cultured at 37°C under 5% of CO_2_, in the appropriate medium containing 10% fetal bovine serum (FBS) and 100 U mL^−1^ penicillin sodium and 100 μg mL^−1^ streptomycin sulfate. Cell type-specific culture conditions include: Dulbecco's modified Eagle's medium (DMEM) supplemented as above plus 1 mM sodium pyruvate for murine Lewis lung carcinoma (LLC) cells; RPMI 1640 medium supplemented as above plus 1 mM, sodium pyruvate and 1 mM HEPES buffer for murine colon carcinoma CT26 cells; RPMI 1640 medium supplemented as above plus 1 mM sodium pyruvate, and 0.04% sodium bicarbonate, and 1 mM non-essential amino acids for progressor (diploid) and regressor (tetraploid) methylcholanthrene (MCA)-induced fibrosarcoma cell lines. The progressor and regressor cell lines used were 9609 and 4862, respectively [18].

### Generation of hyperploid clones

Parental CT26 cells were treated for 48 h with 100 nM nocodazole and then cultured for 2 weeks in drug-free culture medium, followed by cloning of cells characterized by an 8n DNA content, as previously described [23, 31].

### Isolation and culture of engrafted tumor cells

After sacrifice, progressor or regressor tumors were immediately removed and dispersed with 0.25% trypsin-EDTA for 30 minutes at 37°C, followed by mechanical dispersion and cell culture [23].

### Cytofluorometry

For the assessment of cell cycle distribution, harvested cells were fixed in ice-cold 80% (v/v) ethanol and stained with 50 μg mL^−1^ propidium iodide (PI) in 0.1% D-glucose (w/v in PBS) supplemented with 1 μg mL^−1^ RNAse A [32]. Samples were then analyzed by means of an Attune NxT Flow Cytometer (Life Technologies - Thermo Fisher Scientific, Waltham, USA). Statistical analyses were carried out by using the FlowJo^TM^ software (FlowJo LLC, Ashlan, USA), upon gating on the events characterized by normal forward scatter and side scatter values.

### Immunofluorescence microscopy

For eIF2α phosphorylation staining, CT26 cells were seeded onto 384 well cell culture microplates, μClear^®^ (Greiner bio-one, Kremsmünster, Austria), allowed to adapt for 48 h and then fixed with paraformaldehyde (PFA) for 30 min and stored at 4°C in PBS. To assess the staining, cells were washed with PBS, permeabilized with 0.3% Triton X-100 for 10 min at room temperature, RT, and rinsed three times with PBS. Non-specific binding sites were blocked with bovine serum albumin (BSA) for 15 min at RT followed by incubation with the primary antibody (1.4 mg mL^−1^) for 2 h at 37°C. Subsequently, cells were washed three times with PBS and incubated for 30 min in AlexaFluor® 488-conjugated secondary antibody (1:500 in BSA; Molecular Probes-Invitrogen, Eugene, USA). When appropriate, 10 μM Hoechst 33342 (Molecular Probes-Invitrogen) was used for nuclear counterstaining. Images were acquired using an Image Xpress Micro XLS high content imager (Molecular Devices (MDS), Sunnyvale, USA). Images were analyzed with the MetaXpress® software (MDS Analytical Technologies, Sunnyvale, USA).

### Immunoblotting

For immunoblotting, cells were washed with cold PBS at 4°C and lysed following standard procedures. Twenty μg of proteins were separated according to molecular weight on NuPAGE® Novex® Bis-Tris 4–12% pre-cast gels (Invitrogen, Waltham, USA) and electrotransferred to 9 Immobilon polyvinyldifluoride (PVDF) membranes (Millipore, Bedford, USA). Non-specific binding sites were blocked by incubating membranes for 1 h in 0.05% Tween 20 (v/v in TBS) supplemented with 5% non-fat powdered milk or BSA. After overnight incubation at 4°C, primary antibodies (rabbit polyclonal antibodies against eIF2α, and phospho-eIF2α (Ser51)) were detected with the appropriate horseradish peroxidase-labeled secondary antibodies (Southern Biotechnologies Associates; Birmingham; UK) and revealed with the Amersham ECL+ chemoluminescent substrate (GE Healthcare, Little Chalfont, UK). The abundance of β-actin was monitored to ensure equal lane loading.

### Mice

Mice were maintained in specific pathogen-free conditions, and experiments followed the Federation of European Laboratory Animal Science Association (FELASA) guidelines. Animal experiments were in compliance with the EU Directive 63/2010 (protocol 2012_034A) and were approved by the Ethical Committee of Gustave Roussy (Villejuif, France) (CEEA IRCIV/IGR n° 26, registered at the French Ministry of Research). WT C57BL/6 mice were obtained from Harlan France (Gannat, France) and The Jackson Laboratory (Maine, USA), *Rag2^−/−^*γ*_c_^−/−^* mice were obtained from Gustave Roussy (Villejuif, France) and *Rag2^−/−^* mice were obtained from The Jackson Laboratory.

### Tumorigenicity assay and antitumor vaccination

For tumorigenicity experiments, 5 × 10^6^ progressor or regressor cells were inoculated subcutaneously in 200 μL PBS into the lower flank of 6-week-old female C57BL/6, *Rag2^−/−^* and *Rag2^−/−^*γ*_c_^−/−^* mice. Tumors were evaluated weekly using a common caliper. Animals bearing tumors that exceeded 20–25% body mass were euthanatized. C57BL/6 mice that had previously been injected with regressor cells but failed to develop tumors were re-injected with progressor cells and LLC unrelated cell line as a control, in order to establish the possible vaccination quality of regressor cells.

### Immunocytochemistry

Pellets from CT26 parental and hyperploid clones were fixed in formalin for 4 h at RT and then embedded in paraffin. Sections of 4 μm were obtained by means of a RM2245 microtome (Leica Microsystems GmbH, Wetzlar, Germany) and then applied onto histological Polysine®-coated glass slides (Thermo Fisher Scientific). Then samples were deparaffinized in xylene and rehydrated by incubation following 95%, 70%, 50%, 30%, (v/v in PBS) ethanol baths (2 min/bath). HES staining was performed following standard procedures.

### Immunohistochemistry

Samples from cellular pellets and recovered tumors were fixed with 4% PFA for 4 h and then embedded into paraffin. After deparaffinization, eIF2α-phosphorylated staining was performed as follows. Sections of 5 μm were stained with a monoclonal anti-phospho-eIF2α (Ser51) (3597, Cell Signaling) on a Discovery Ultra automated immunostainer (Ventana, Tucson, USA). Antigen retrieval was performed by incubating slides in EDTA buffer (pH 8.0) for 32 min at 95°C. And then the antibody was incubated for 1 h at 37°C at the final concentration of 4 μg mL^−1^. Finally, the samples were counterstain with hematoxylin II for 12 min followed by Bluing Reagent for 8 min (Ventana). After staining, images were acquired with a Virtual Slides microscope VS120-SL (Olympus, Tokyo, Japan), 20X air objective (0.75 NA).

### Morphometric analysis of paraffin-embedded excised tumors

Images were extracted from the original VSI-coded files and converted to the TIFF file format by means of the VSI-Reader tool developed and implemented in the Fiji software (http://fiji.sc/Fiji) by the BioImaging and Optics Platform of EPA (http://biop.epfl.ch/TOOL_VSI_Reader.html). These images were thereafter analyzed by means of a morphometric analysis algorithm that we developed in R (https://www.r-project.org/) using the EBImage processing toolbox.

The code is freely available and can be downloaded on https://fileshare.gustaveroussy.fr:443/easyshare/fwd/link=lMfqvir41hZeTLfQFT2QGD. Briefly, in order to measure nuclear dimension and cytoplasmic eIF2α-specific signal, the red and blue components of the image were extracted and enhanced using a log transformation. Resulting images were combined and segmented into nuclear and cytoplasmic masks (the latter generated by defining a 7 pixel ring around the nuclear region), with which the number of pixels in the nuclear area and the pixel intensity of the cytoplasmic (only perinuclear, not nuclear) eIF2α signal were measured.
